# Our experience with home self‐assessment of speech recognition in the care pathway of 10 newly implanted adult cochlear implant users

**DOI:** 10.1111/coa.13307

**Published:** 2019-03-06

**Authors:** Feike de Graaff, Elke Huysmans, Birgit Philips, Paul Merkus, S. Theo Goverts, Sophia E. Kramer, Cas Smits

**Affiliations:** ^1^ Otolaryngology ‐ Head and Neck Surgery, Ear and Hearing, Amsterdam Public Health Research Institute, Amsterdam UMC Vrije Universiteit Amsterdam Amsterdam The Netherlands; ^2^ Cochlear Technology Centre Belgium Mechelen Belgium

**Keypoints**

- The number of cochlear implant (CI) users has grown rapidly, resulting in an increased workload for CI centres and a need for new and innovative ways to provide healthcare to users of a CI.
- A telehealth application was developed with a functionality to self‐administer speech recognition tests at home, which was evaluated in 10 newly implanted patients.
- Speech recognition in quiet and in noise improved steadily during the first few weeks of rehabilitation, after which it stabilized.
- The home tests provided a good alternative to testing in the clinic for newly implanted patients who were able and willing to perform part of their CI care from home and felt confident in using the technology required.
- Frequently administered speech recognition self‐tests provide fine‐grained progress details which enable clinicians to monitor their CI user’s speech recognition ability over time without the need for users of a CI to visit the clinic.

## 1 INTRODUCTION

The number of newly implanted cochlear implant (CI) patients is increasing rapidly, due to changing regulations, expanding candidacy criteria and technical improvements in CIs. This results in an increased workload for CI centres and opens the door for new and innovative ways to provide healthcare to users of a CI. Rehabilitation after cochlear implantation is very demanding and time consuming for newly implanted patients. It requires frequent and long visits to the clinic within the first year after implantation. The sound processor is fitted or fine‐tuned during these visits and auditory training is provided. Counselling on how to use and maintain the CI is provided as well as speech recognition testing. Speech recognition is an important outcome measure during rehabilitation and is typically assessed in the clinic by a clinician with calibrated equipment.

Within the “Supporting Hearing in Elderly Citizens” project, we developed a telehealth application, the MyHearingApp. The application comprises a user interface for a tablet computer with, among other functionalities, a functionality to self‐administer speech recognition tests at home. We demonstrated that experienced users of a CI were able to perform self‐administered speech recognition tests at home and that the home tests provide a valid alternative to testing in the clinic.^1^, ^2^ The MyHearingApp usability was assessed and observed to be satisfactory in a random group of 16 senior (60+) experienced users of a CI. They ranked the ability to perform home tests as the most relevant functionality.^3^

The main objective of the current study was to investigate the use and feasibility of the MyHearingApp self‐test functionality in care‐as‐usual of newly implanted patients. We evaluated whether newly implanted patients would comply with instructions to repeatedly perform speech recognition tests at home, and we collected their experiences with the self‐test. Another objective was to describe the progress in speech recognition performance during the first 3 months of rehabilitation in a more fine‐grained manner than in current rehabilitation care.

## 2 MATERIALS AND METHODS

### 2.1 Ethical considerations

The study was approved by the Medical Ethics Committee of VU University Medical Center Amsterdam. The participants enrolled into the study voluntarily and provided informed consent.

### 2.2 Study participants

Ten consecutive newly implanted adult patients (seven males; three females) participated in this study (Table 1). They were postlingually deaf (onset of severe hearing impairment after the age of 7 years), were unilaterally implanted with the Cochlear™ Nucleus® CI24RE implant with Contour Advance Electrode and used the Cochlear™ Nucleus® CP910 sound processor. No selection criteria were set in terms of computer experience.

**Table 1:** Demographic characteristics, total number of scheduled speech recognition tests and number of tests performed by each participant

Participant	Gender	Age (y)	Total number of scheduled tests^a^	Tests performed	
				Speech recognition in quiet	Speech recognition in noise
S1	F	77	22	17 (77%)	17 (77%)
S2	M	64	22	19 (86%)	19 (86%)
S3	M	78	24	23 (96%)	23 (96%)
S4^b^	M	78	‐	‐	‐
S5^b^	M	74	‐	‐	‐
S6	M	67	22	20 (90%)	20 (90%)
S7	F	33	20	14 (70%)	15 (75%)
S8	M	49	20	8 (40%)	8 (40%)
S9	M	67	22	19 (86%)	18 (82%)
S10	F	20	22	5 (23%)	5 (23%)
Total			174	125 (72%)	125 (72%)

F, Female; M, Male.

a The total number of scheduled speech recognition tests for each participant is different, because of differences in the rehabilitation schedule.

b S4 and S5 withdrew from the study prematurely.

### 2.3 Procedures

The study was conducted according to a prospective within‐subject design in conjunction with care‐as‐usual. Self‐tests were done using a tablet computer (Lenovo Thinkpad 10; Lenovo International Cooperative U.A., Amsterdam, The Netherlands) and an audio cable that directly presented stimuli to the sound processor. The mixing ratio of the sound processor was set to “accessory only,” ensuring that participants only received sound coming from the audio cable. The participants received the tablet computer with the MyHearingApp in the week after activation of their sound processor. Participants were instructed to assess their speech recognition at home twice weekly during the first 3 months of rehabilitation. The tests were scheduled by a clinician and subsequently appeared in the task list in the MyHearingApp. Participants were allowed to perform the tests on different days, as long as they were performed prior to the next scheduled test. Otherwise, tests were no longer accessible to the participant.

After 3 months, participants returned the tablet computer at their regular visit to the clinic and were asked to complete a questionnaire to elaborate on their experiences with the self‐test functionality (see Table 2 for details). Speech recognition was assessed again in the clinic with the tablet computer after 6 months of rehabilitation.

**Table 2:** Specific questions from the questionnaire addressing the home self‐assessment of speech recognition with the individual and mean scores

Question	Scores								
	S1	S2	S3	S6	S7	S8	S9	S10	Mean
1 How clear do you find the graph with the speech recognition in quiet results? Very unclear (0)—Very clear (10)	10	8	10	8	9	7	8	5	8.1
2 How clear do you find the graph with the speech recognition in noise results? Very unclear (0)—Very clear (10)	10	8	10	6	8	7	8	7	8.0
3 How useful do you think it is to see the progress in speech recognition over time? Not useful (0)—Very useful (10)	10	8	9	8	10	9	9	6	8.6
4 How reliable do you think the results of the home tests are compared to the tests in the clinic? Very unreliable (0)—Very reliable (10)	6	7	7	8	9	8	8	7	7.5
5 How useful do you think it is to be able to self‐assess your speech recognition at home? Not useful (0)—Very useful (10)	6	9	10	8	10	8	8	7	8.3
6 What do you prefer for speech recognition testing in the future? Always at home (1) Frequently at home, sometimes in the clinic (2) Frequently in the clinic, sometimes at home (3) Always in the clinic (4)	2	2	2	1	3	4	3	3	

### 2.4 Speech recognition tests

Speech recognition in quiet was assessed using monosyllabic words with a consonant‐vowel‐consonant (CVC) structure, pronounced by a female Dutch speaker.^4^ Lists of 12 CVC words, each word containing three phonemes, were presented at 65 dB SPL. The score was calculated as the percentage of phonemes recognised correctly. The response on the first word was not included in the calculation of the score.

Speech recognition in noise was assessed with the digits‐in‐noise test.^5^, ^6^ The test estimates the speech reception threshold (SRT) via an adaptive procedure using series of digit‐triplets (eg 6‐5‐2) presented against a background of continuous steady‐state speech‐shaped masking noise. The SRT represents the signal‐to‐noise ratio (SNR) at which the listener recognises 50% of the digit‐triplets correctly. Lower SRTs represent better speech recognition in noise.

At the start, two CVC tests were listed in the task list. Once the participant reached an average phoneme‐correct score of at least 40% on the two CVC tests, they were instructed to perform one digits‐in‐noise test as well. Hereafter, participants performed two CVC tests and one digits‐in‐noise test. The task list took <10 minutes to complete.

Test results were made visible in the MyHearingApp for the participant in two separate graphs, for speech recognition in quiet and in noise. The graphs showed new and previous results (Figure S1). For speech recognition in quiet, the mean score of two CVC tests with the standard deviation was plotted. For speech recognition in noise, the SRT with standard error of measurement (1.1 dB, based on previous research^6^) was plotted. As the SRT may be difficult to interpret for lay people, we opted for an interval scale with categories varying from <−7.5 to >7.5 dB SNR (Table S1). The detailed test results were remotely visible for clinicians.

## 3 RESULTS

### 3.1 Feasibility and compliance

One participant withdrew after 2 weeks (S5) and another one after 2 months (S4). Both withdrew because they felt that they had insufficient computer knowledge and skills to perform tests at home. The remaining eight participants performed approximately 75% of the scheduled tests (Table 1). Two participants performed less than half of the scheduled tests, because of time constraints and lack of motivation (S8) or fatigue and tinnitus (S10).

### 3.2 Speech recognition

**Figure 1:**
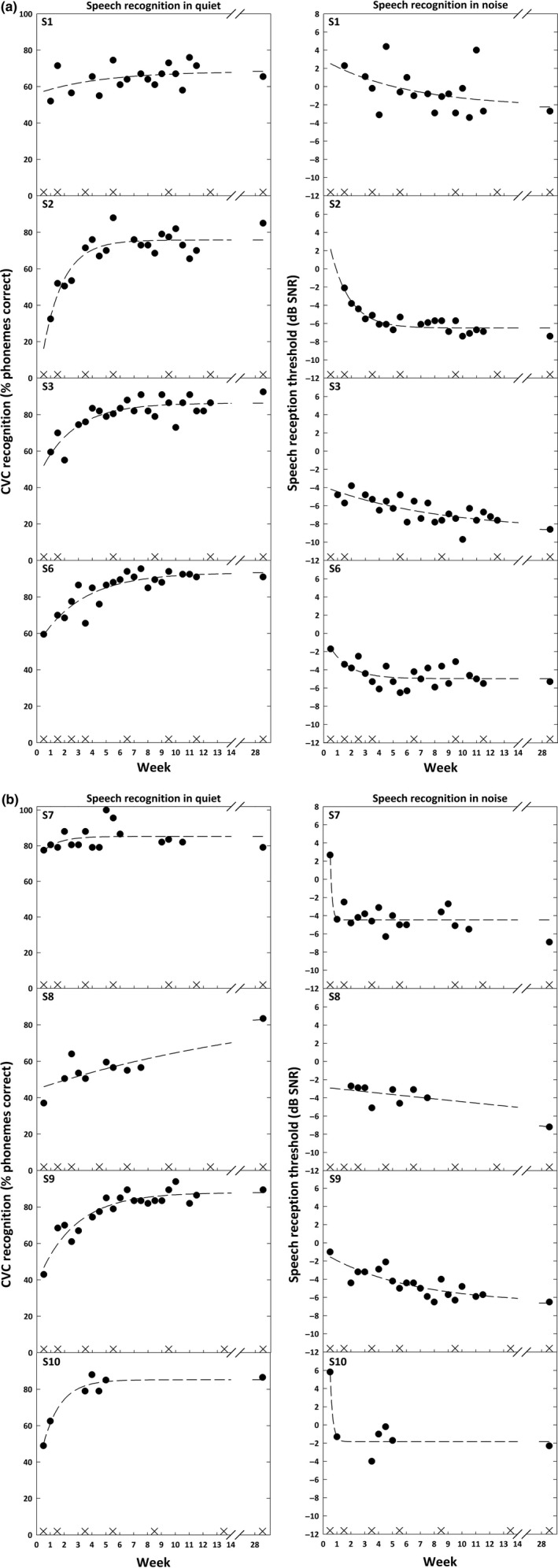
Speech recognition in quiet and speech recognition in noise scores, assessed twice a week during the first 3 mo of rehabilitation and again after 6 mo. The crosses on the *x*‐axes represent fitting appointments with the audiologist in the clinic. The dashed lines show exponential fits to the data points

The speech recognition test results are shown in Figure 1. The dashed lines show exponential fits to the data points. All participants, except two (S1 and S7), showed a clear steady increase in speech recognition in quiet and in noise during the first 4‐5 weeks. Thereafter, speech recognition stabilized. Half of the participants (S3, S6, S7, S9) reached a score of 80% of phonemes correct or higher 3 months after activation. One participant (S7) even reached 100% speech recognition in quiet in week 5.

### 3.3 Questionnaire

Responses to the questionnaire are listed in Table 2. Overall, participants gave a mean score of 8 for the possibility to self‐assess their speech recognition. Half of participants reported to prefer home testing over testing in the clinic. Presentation of the test results was considered useful and clear and helped to motivate participants to improve their performance further. Most of the participants considered the results reliable, except one (S1), mainly due to variation in her test results (Figure 1).

## 4 DISCUSSION

The purpose of this study was to evaluate the use and feasibility of self‐administered home speech recognition testing in newly implanted patients. The results show that home tests might be suitable for approximately 80% of newly implanted patients. Because we included 10 consecutive newly implanted patients without any selection criteria, we think that our participants are representative of newly implanted patients, despite the small number. In current care, patients visit our clinic nine times during the first 3 months for a total of 19 hours. For those patients eligible for home self‐assessment, a large reduction in visits and time seems achievable while information about speech recognition progress is even more detailed for clinicians than currently.

### 4.1 Clinical applicability

To our knowledge, this is the first study showing progress in speech recognition over the first 3 months of rehabilitation in such detail. The data reveal improvements in speech recognition over time, without a clear relation to fitting appointments with an audiologist. Detailed progress information has not been available to clinicians before, because in care‐as‐usual speech recognition is assessed only once or twice in the equivalent period. Detailed progress information enables clinicians to identify—at a much earlier stage—for whom the level of auditory training would need to be intensified (eg for S1 with unsatisfactory progress in the first weeks). The detailed results also indicate for whom visits to the clinic would become unnecessary or could be reduced (eg for S2, S3, S6, S9 for whom progress was satisfactory). The current data are promising and indicate that there is more potential for home self‐assessment. It could be used to try out different settings of the sound processor at home while simultaneously allowing clinicians to examine effects on speech recognition without the need for additional visits to the clinic. To illustrate, the sound processor could be programmed with two different programs and the patient could perform multiple tests at home while acclimatising to the new settings.

## 5 CONCLUSION

In conclusion, home self‐testing has the potential to change and improve the CI care pathway substantially, eventually leading to a significant reduction in the required number of visits of patients to the clinic. Not only would this result in cost‐ and time savings for both clinics and patients, it would even improve the quality and richness of data obtained during rehabilitation. A reduction in the number of technical operations (eg digital streaming of stimuli) might improve the usability of the home tests for even more newly implanted patients.

## CONFLICT OF INTEREST

The authors report no conflicts of interest.
