# Supplementary figures and images for: Our experience with home self‐assessment of speech recognition in the care pathway of 10 newly implanted adult cochlear implant users
Source: Clin Otolaryngol. 2019 Mar 6;44(3):446–51. doi: 10.1111/coa.13307 (PMC6850507; doi:10.1111/coa.13307)

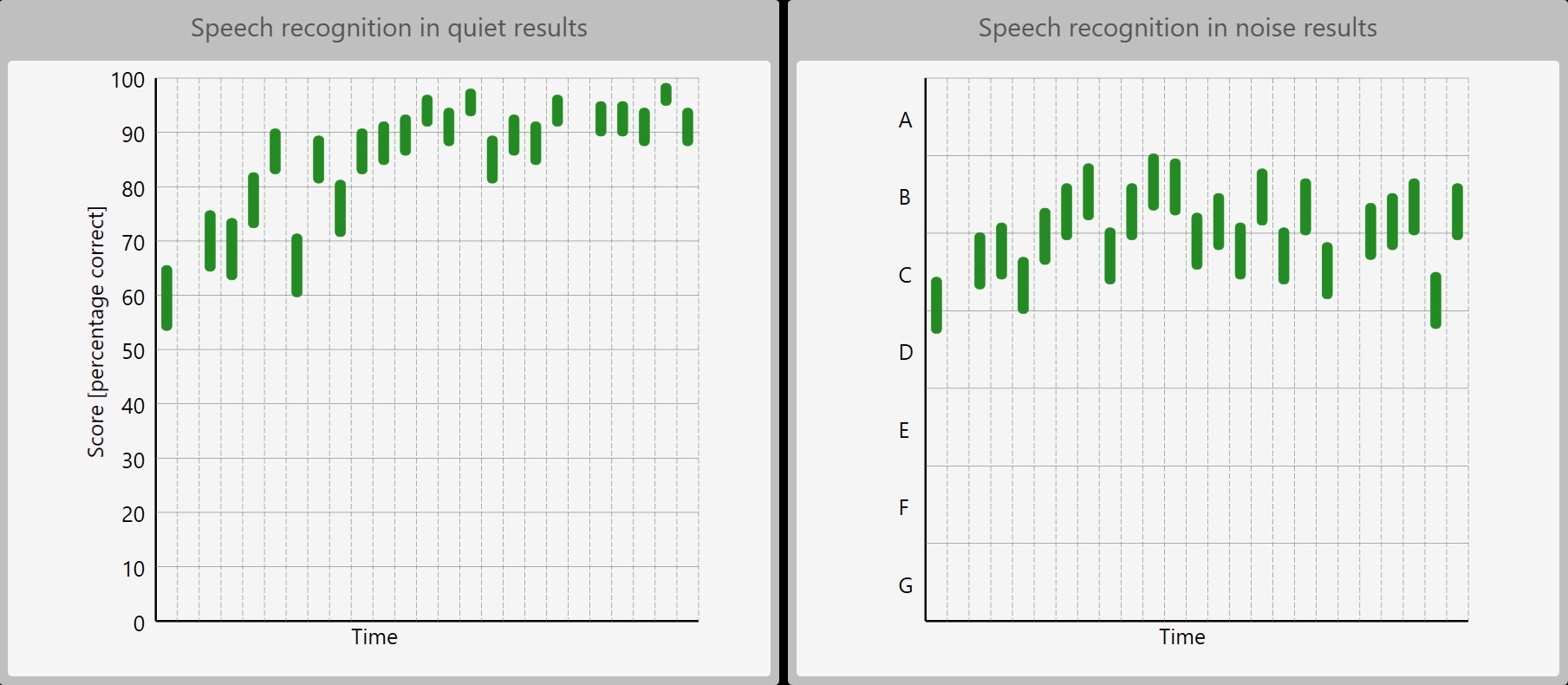

Supplement: Supplementary file 1 [file COA-44-446-s001.jpg]
