# Supplementary material for: Our experience with home self‐assessment of speech recognition in the care pathway of 10 newly implanted adult cochlear implant users
Source: Clin Otolaryngol. 2019 Mar 6;44(3):446–51. doi: 10.1111/coa.13307 (PMC6850507; doi:10.1111/coa.13307)
Supplement: Supplementary file 2 [file COA-44-446-s002.docx]

Table 1. Details of the speech reception threshold (SRT) categories in dB SNR that were used to present the results of the digits-in-noise test to the participants.

| **Category A**  < -7.5 dB SNR  (reference category for NH adults; Smits et al., 2013) |
| --- |
| **Category B**  -7.5 to -4.5 dB SNR |
| **Category C**  -4.5 to -1.5 dB SNR |
| **Category D**  -1.5 to 1.5 dB SNR |
| **Category E**  1.5 to 4.5 dB SNR |
| **Category F**  4.5 to 7.5 dB SNR |
| **Category G**  > 7.5 dB SNR |
